# Investigation of the physical and psychosocial outcomes after living kidney donation - a multicenter cohort study (SoLKiD - Safety of Living Kidney Donors)

**DOI:** 10.1186/s12882-018-0871-z

**Published:** 2018-04-10

**Authors:** Barbara Suwelack, Viktoriya Wörmann, Klaus Berger, Joachim Gerß, Heiner Wolters, Frank Vitinius, Markus Burgmer, for the German SoLKiD consortium, Anja S. Mühlfeld, Fabian Halleck, Klemens Budde, Petra Reinke, Anett Sefrin, Frank Vitinius, Roger Wahba, Christian Hugo, Mirian Opgenoorth, Magdalena Woznowski, Anita Hansen, Andreas Kribben, Oliver Witzke, Steffen Platschek, Ingeborg A. Hauser, Rolf Weimer, Lucy Rainer, Karl Weigand, Gerit Theil, Sylvia Kroencke, Martina Koch, Mario Schiffer, Frank Lehner, Martin Zeier, Claudia Sommerer, Thomas Rath, Georgios Papadopoulos, Thorsten Feldkamp, Astrid Fülbier, Martin Nitschke, Jessika Schlieter, Beate Schamberger, Jens Lutz, Kai Nowak, Bernd Krüger, Manuela Kirchner, Kai Lopau

**Affiliations:** 1 Department of Internal Medicine D, Transplant Nephrology, University Hospital of Münster, Waldeyerstr.1, 48149 Münster, Germany; 2 Department of Psychosomatics and Psychotherapy, University Hospital of Münster, Domagkstr. 22, 48149 Münster, Germany; 3 Institute of Epidemiology and Social Medicine, University of Münster, Domagkstr. 3, 48129 Muenster, Germany; 4 Institute of Biostatistics and Clinical Research, University of Münster, Schmeddingstr. 56, 48149 Münster, Germany; 5 Department of General and Visceral Surgery, University Hospital of Münster, Albert-Schweitzer-Campus 1, 48149 Muenster, Germany; 6 Department of Psychosomatics and Psychotherapy, University Hospital of Cologne, Kerpener Str. 62, 50937 Cologne, Germany

## Abstract

**Background:**

Over the last years, living kidney donation (LKD) has been established for patients with endstage renal failure as an alternative to post mortem donation, which is limited by organ scarcity and long lasting waiting periods. From an ethical perspective, the increase in LKD requires that donors’ physical, psychological, and social harm has to be minimized as much as possible and the risk should not exceed the generally expected consequences of nephrectomy. Despite of numerous, mainly retrospective studies about the postoperative outcome of LKD over the last years from different countries, it becomes apparent that there is a lack of comprehensive prospective multicenter research in this field worldwide. Therefore, the main aim of the study is to examine the physical and psychosocial outcome of living kidney donors in a prospective design before and after transplantation in an interdisciplinary approach (surgery, nephrology, psychosocial medicine).

**Methods/design:**

The goal of the study is to investigate such aspects as the impact of gender- and age-specific factors on LKD outcome, donor outcome in correlation to the health status of the recipient, the medical and psychosocial risk of a healthy subject undergoing the LKD procedure. The study is carried out as a nationwide multicenter study. All adult living kidney donors with sufficient knowledge in the German, Russian, or Turkish language, informed consent, and place of residence in Germany are included. In a naturalistic design (cohort study), clinical data and self-report measures (questionnaires) of 320 donors are collected before and 8 weeks, 6 and 12 months after donation. Primary outcome parameters are the kidney function (estimated GFR) and the quality of life (SF-36) of the donor. Secondary outcome parameters are data about physical (e.g., wound healing, blood pressure) and psychosocial (fatigue, depression, anxiety, somatization) outcome after donation.

**Discussion:**

Previous studies on the postoperative outcome of living kidney donors have methodological limitations and/or were carried out in countries with different healthcare systems, e.g. United States, Norway, Canada, United Kingdom. Thus, results cannot be generalized and are not particularly applicable to the risks of mainly caucasian living kidney donors in the German healthcare system. The study design overcomes these disadvantages in that it provides a prospective multicenter design.

**Trial registration:**

German Clinical Trials Register DRKS00006552 (22 September 2014).

## Background

For patients with endstage renal disease (ESRD), transplantation is currently the only treatment to regain an adequate renal function. Post mortem donation normally requires a long waiting time for the transplant recipient, due to the limited availability of organs. The actual waiting time in Germany is 6–7 years according to the German Organ Transplantation Foundation (Deutsche Stiftung Organtransplantation, https://www.dso.de/). Therefore, LKD has become an established alternative in recent years, which has many advantages for the kidney recipient. The transplantation can be done preemptively and is associated with a better rate of survival of the transplanted kidney as well as the organ recipient [1, 2]. Quality of life (QoL) and life expectancy of the kidney recipient are improved and the occurrence of cardiovascular complications is reduced [3, 4]. Furthermore, a faster rehabilitation and a better reintegration into the daily activities and the occupational life contribute to reduce healthcare costs by € 280.000 compared to continuing dialysis treatment [5]. Worldwide the percentage of LKDs among all kidney transplantations varies widely due to the scarcity of organs from post mortem donors (KDIGO Guideline Evidence Report) [6]. In Germany, approximately 30% (living donors *n* = 645 / post mortem *n* = 1550) of all performed kidney transplantations were done with a living donor organ in 2015 (statistics.eurotransplant.org).

The German transplantation law requires that living donors should not be put at excessive risks due to the operation or its consequences. Relatively few published studies examined the surgical, general health, and psychosocial risks of donors following donation, most being retrospective in design. Older studies did not find an increased risk of advanced renal failure or of a relevant decline in glomerular filtration rate (GFR) in kidney donors compared to the general population [7–10]. In contrast, recent publications from registers in the US and in Norway reported a less favorable renal outcome among living kidney donors compared to a control group of potential donors from the healthy population [11, 12]. The position paper of the European Committee on Organ Transplantation (CD-P-TO) criticized these studies because of their methodological limitations and listed a series of evidence, which contradict the results of both studies [13]. With relation to comorbidities, studies examining other outcomes, such as the impact of hypertension [14–16] or obesity [17] are scarce and also report conflicting results.

There are no prospective randomized multicenter studies on the psychosocial long-term risks and consequences for donors. Frade et al. examined QoL in donors before and after (18,8+/− 12,8 months) donation and found only one significant improvement after donation of social functioning scores (from 79.1 to 89.8). The other SF-36 subscales remained unchanged [18]. Another study showed that living kidney donors after donation (on average 32 months) scored significantly better among the SF-36 scales “social functioning,” “bodily pain,” and “vitality” compared to the controls (healthy population) or to patients with renal tumors [19]. Some other studies, however, found that QoL was significantly reduced in the areas of physical [20–25] or mental health related [26, 27] components of QoL within the first post-operative period (up to 6 months). Most of these reductions were no longer found, however, at later measurement points. Some studies showed increased pain and fatigue within the first weeks after surgery and a higher incidence of mental disorders during the first 12 months postoperatively [20, 26, 28]. Important limitations of prior prospective studies included small sample sizes, monocentric conduction and the neglecting of the health status of the recipient as possibly influencing the donor’s well-being. Further limitations include a lack of gender-specific aspects [29, 30] and the problem of limited transferability of results to other countries due to differences in the healthcare systems.

Aim of the SoLKiD study (“Safety of Living Kidney Donors”) is to examine physical and psychosocial outcomes of living kidney donors based on a prospective multicenter design, an integration of relevant disciplines (surgery, urology, internal medicine, nephrology, psychosocial medicine), a detailed assessment of the psychosocial status at several time points, and additionally taking the recipient’s health status into account.

## Methods/design

### Study centers

The study is carried out at 20 transplant centers in Germany. Participating centers are the University Hospitals of RWTH Aachen, Campus Charité Berlin Mitte, Campus Charité Berlin Virchow, Dresden, Düsseldorf, Essen, Frankfurt, Giessen, Hamburg-Eppendorf, Heidelberg, Halle (Saale), Schleswig-Holstein Campus Kiel, Schleswig-Holstein Campus Lübeck, Köln, Mainz, Mannheim, Münster, Würzburg, Westpfalz-Klinikum Campus Kaiserslautern, and Hannover Medical School. Donor-recipient pairs are recruited in the respective outpatient departments of the hospitals after signing informed consent. Study coordination is done at the University Hospital of Münster. The study is performed in accordance with the declaration of Helsinki and with the local ethics committees of all participating centers.

### Participants

Inclusion and exclusion criteria for living kidney donors are listed in Table 1.

**Table 1:** Inclusion and exclusion criteria of the SoLKiD study for living kidney donors

Inclusion criteria	• Living kidney donors before upcoming nephrectomy
	• Age > 17 years
	• Informed consent
	• Native language German, Turkish, Russian
Exclusion criteria	• Refusal to participate
	• Not living in Germany
	• Lack of reading comprehension in the native language

As a common consensus between the participating centers, the Russian-speaking and Turkish-speaking donors are included in the study in addition to German-speaking donors, as they constitute a representative part of the population in Germany and because validated questionnaires in Turkish and Russian language are available. The study started in 2014 and included 320 donors for the baseline assessment prior to transplantation, corresponding to approximately 50% of the annually conducted living donations in Germany. With an expected follow-up rate of > 80%, approximately 260 participants can be followed up to 1 year after donation.

### Measurement time points

**Fig. 1:**
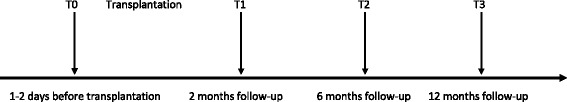
Time points of measurement in the SoLKiD study

Living kidney donors and the associated organ recipients are examined at four time points (T0, T1, T2, T3) before donation and during the regular follow-up visits required by the transplant centers. All data for the baseline assessment (T0) is collected immediately (1–2 days) before the donation in order to avoid in the case of the earlier baseline assessment (e.g. during the preparation process for the donation) the possible donors’ concern that they might be considered as not suitable for donation because of their answers. The data collection for all time points corresponds with the clinical routine process and is imbedded into the usual care practice of LKD. The time points of measurement of the study are shown in Fig. 1.

### Study measures: Primary and secondary outcome variables

Data are collected by the transplant centers within the usual donor and recipient preparation and care. They include laboratory values ​​(serum creatinine, eGFR, proteinuria, blood sugar levels), and clinical data (blood pressure, weight, surgical complications). In addition, further information, in particular psychosocial status variables, are collected by means of questionnaires at four time points. Questionnaires used in the study are listed in Table 2.

**Table 2:** Questionnaires used in the SoLKiD study for living kidney donors

Questionnaire	Short description	Scales
SF-36 [45, 46] (Short-Form 36-Item Health Survey)	Standardized, generic instrument for the detection of health-related quality of life in the general population and in different patient groups. 8 dimensions can be conceptually summarized in two components: “physical health” and “mental health”.	Physical functioning, physical role function, bodily pain, general health, vitality, social functioning, emotional role function, and mental health; summary scales: physical health summary score, mental health summary score
MFI [47, 48] (Multidimen-sional Fatigue Inventory)	Standardized self-assessment tool that measures perceived tiredness (fatigue) in extent, nature, and intensity. The values can be compared inter- and intra-individually.	General fatigue, physical fatigue, reduced activity, reduced motivation, mental fatigue
PSS-10 [49] (Perceived Stress Scale)	Instrument for the measurement of the subjective stress rating and evaluation of the global perceived stress. It measures a degree of frequency, how the answerers grade their lives as unpredictable, uncontrollable, and overburdening.	Global degree of frequency of perceived stress
PHQ-D Short Form [50] (Patient Health Questionnaire)	The questionnaire was developed to facilitate the detection and diagnosis of the most common mental disorders in primary medicine. In addition to questions about mental disorders, there are also items for psychosocial function ability, stressors, critical life events and - for women - menstruation, pregnancy, and childbirth. In addition to the categorical diagnosis of mental disorder, severity ratings for the areas depression, somatic symptoms and stress can be provided (continuous diagnostics).	The short form, which is used in the present study, refers to the following scales: Depression (PHQ-9); Anxiety (GAD-7); Somatization (PHQ-15). For these scales, the formation of a separate scale for each point value (scores) for measuring the severity is possible.
CERQ [51, 52] (Cognitive Emotion Regulation Questionnaire)	Instrument for detecting the different components of habitual cognitive emotion regulation. It comprises nine dimensions, which measure the possible mental responses to an aversive event and thus triggered emotions.	Catastrophizing, self-blame, rumination or focus on thought, positive reappraisal, positive refocusing, refocusing on planning, other-blame, putting into perspective, acceptance

Estimated GFR (eGFR) using the CKD-EPI creatinine equation [31] of the donor and the physical and mental summary scores of the SF-36 are defined as primary outcome variables. All other outcomes of the physical and mental health status of the donor are used as secondary outcomes.

Psychosocial consequences for a donor can either be assessed as a reduction in the mental health score of the SF-36, or as an increase in depression or anxiety scores, or as an increase in scores for somatization (PHQ-D), change in psychosocial functioning and increased stress (PSS-10) or fatigue (MFI). Additionally, at baseline different components of a habitual cognitive emotion regulation scale (CERQ) are assessed, enabling a control of the relationship between the present ability of adequate emotion regulation and postoperative psychosocial consequences in donors.

Psychosocial variables which reflect the current mental state of donors additionally include the subjective attitude towards donation and the assessment of donor-specific relationship aspects to the recipient. This includes, for example, if a donor would donate again, or if he/she felt him/herself to be pushed towards donation by others. All questionnaires are offered either in German, Russian or Turkish validated versions. Information is collected independently from donor and from recipient to avoid a two-way influence during the assessment. The standardized assessment form with the clinical data of donors and recipients plus the donor questionnaires are sent in a pseudonymized manner to the data management center in Münster at all four time points.

### Statistical analysis

Statistical analyses will be performed according to the principles of the ICH-guideline E9 “Statistical Principles for Clinical Trials” using standard statistical software.

A detailed description of the study population will be given using summary statistics such as mean and standard deviation, median and quartiles, or absolute and relative frequencies, as appropriate.

Each of the three co-primary outcomes, i.e. the donors’ eGFR and QoL according to the SF-36 “physical health” and “mental health” summary score will be analyzed using a *Mixed Linear Model*. This method is chosen in order to account for repeated measurements of individual donors’ outcomes over time (longitudinal data). One important advantage of the *Mixed Model* approach is that missing values in outcomes are handled in a way that prevents biased results. In the established models, all relevant independent variables will be included that impact the respective outcome, first of all the baseline values of the respective outcomes. Furthermore prognostic factors as gender, age, and education will be included in the model in order to adjust for confounding. Basic model assumptions such as the Gaussian distribution of model residuals will be checked using graphical methods, including histograms and quantile-quantile plots. If necessary, outcome variables will be transformed appropriately, applying square-root, logarithmic or *Box-Cox* transformations. Models will be fitted using *Restricted Maximum Likelihood* methods. Model selection will be performed using stepwise variable selection methods based on *Akaike’s* information criterion. Final models will be validated by diagnostic checks including the calculation of goodness of fit statistics and the examination of the randomness of model residuals. The model’s predictive performance will be checked using *cross-validation*.

In the primary statistical analysis, the following hypothesis test problems are established in an analogous manner in case of each of the three co-primary outcomes eGFR, SF-36 “physical health” and “mental health” summary score. Denote μ_i_ (*i* = 1,2,3) the expected values of the respective outcome at three consecutive follow-up visits T1, T2, and T3. The global null hypothesis H_0_:μ_1_ = μ_2_ = μ_3_ is established and tested against the alternative H_1_:∃(i,j)∈{1,2,3}: μ_i_ ≠ μ_j_ in order to detect any kind of mean changes over time. Beyond the global null hypothesis, pairwise comparisons of expected values μ_i_ (i = 1,2,3) are performed by testing the null hypotheses H_0_^(12)^: μ_1_ = μ_2_, H_0_^(13)^: μ_1_ = μ_3_ und H_0_^(23)^: μ_2_ = μ_3_ against the corresponding two-sided alternatives. The overall multiple significance level across the three co-primary outcomes is set to α = 0.05. In order to keep this significance level, Bonferroni adjustment is applied. Each of the three co-primary outcomes is assigned the local significance level α_loc_ = 0.05/3 = 0.0167, i.e., α_loc_^eGFR^ = 0.0167, α_loc_^SF36phys^ = 0.0167 and α_loc_^SF36mental^ = 0.0167. Within each of the three co-primary outcomes, the following multiple testing strategy is applied, that is derived from the closed test principle. Denote the calculated *p*-values of the pairwise null hypotheses H_0_^(ij)^: μ_i_ = μ_j_ (i,j∈{1,2,3}) p_ij_ and the p-value of the global null hypothesis H_0_:μ_1_ = μ_2_ = μ_3_ p_123_. Any specific pairwise null hypothesis H_0_^(ij)^ is rejected if and only if both p_ij_ ≤ 0.0167 and p_123_ ≤ 0.0167. This multiple testing strategy provides strong control of the family-wise type I error rate α_loc_ = 0.0167. Each of the above null hypotheses will be tested using *Wald* type tests of the respective contrasts in the final established *Mixed Linear Model*. Point estimators of all contrasts will be supplemented by (two-sided) confidence intervals. The primary efficacy analysis provides confirmatory statistical evidence. Secondary outcomes will be analyzed using descriptive and inferential statistical methods. Appropriate tests will be applied for pre-specified hypotheses. Point estimates and associated 95% confidence intervals will be provided.

Further exploratory and safety analyses will be performed. In exploratory and safety analyses, no correction for multiplicity will be applied. An overall or multiple significance level is not determined and cannot be calculated. Results are regarded noticeable in case *p* ≤ 0.05, and are interpreted, accounting for the relatively low level of scientific evidence.

### Sample size calculation

The necessary number of study participants follows from the anticipated effects in the three co-primary outcomes. In Gossmann et al. [7], the observed mean eGFR was 92 mL/min/1.73m^2^ before and 71 mL/min/1.73m^2^ after donation (relative difference |Δrel| ≈ 23%), each with a standard deviation of 15–20 mL/min/1.73m^2^. Similar results were obtained in the study by Ibrahim et al. [9]. In Kroencke et al. [21], average SF-36 “physical health” summary scores were observed at successive measurement time points, that vary between 51 and 56 units (|Δrel| ≈ 10%), each with a standard deviation of up to 8 units. The SF-36 “mental health” summary score amounted to around 53 units and relatively stable in time, with standard deviations of 6.3, 8.9 and 7.7 units at the three consecutive measurement time points. Similar results were obtained in the retrospective study of Wiedebusch et al. [32], who reported SF-36 summary scores of 51–52 units with a standard deviation of 9 units 6 months after surgery. In case of all outcomes, measures obtained from an individual study participant at successive measurement time points are assumed to be positively correlated. A total number of 320 pairs of kidney donors and recipients will be included in the study, resulting in 2 × 320 = 640 study participants. A number of 200 pairs of kidney donors and recipients is expected to provide evaluable data. With this sample size, the above reported differences in the co-primary outcomes eGFR and SF-36 “physical health” summary score can be detected with a power of > 90% in the primary statistical analysis. For the third co-primary outcome SF-36 “mental health” summary score, possible mean differences between successive measurement time points in the order of 3 units (|Δrel| ≈ 6%) can be detected with a power of > 85%. Power calculations were performed using the SAS software.

## Discussion

The aim of the SoLKiD study is to investigate the physical and psychosocial outcomes of living kidney donors during a 12-months course after donor nephrectomy in a prospective multicenter design. It aims to answer the question, which health risks might arise from the elective removal of one kidney from a healthy donor. The interdisciplinary approach enables the assessment of a broad number of physical and psychosocial health status outcomes in order to provide a comprehensive overview of the consequences and the natural course after LKD. Studies on the physical consequences of LKD show divergent results. They reported, on the one hand, an age-dependent decrease of the eGFR of up to 30% within 10 years after donation especially in female donors [7–10]. On the other hand, they did not find a higher rate of renal insufficiency, compared to the general population [9]. However, these studies were mostly based on young and healthy donors. Register studies in the US and Norway showed reduced life expectancy and higher rates of ESRD than in the healthy population basically able to donate [11, 12]. Regarding arterial hypertension as the most important risk factor in the general population for cardiovascular morbidity and mortality, about 15% of donors develop it after kidney donation (in Germany up to 10%) [14, 33]. Data from the Norwegian health registers showed a prevalence of hypertension 5 years after donation of about 27% [15]. In contrast, another study did not find an increased prevalence of hypertension in donors compared to their non-nephrectomized twins [16]. Such differing results could be explained by varying observation times, in the study by Mjoen et al. 5 years after LKD, in the study by Najarian et al. 20 years after LKD [15, 16]. Some studies found an increased cardiovascular mortality especially in the subgroup of older donors with declining eGFR [34, 35]. Generally speaking, findings about the increased mortality in living kidney donors reported above cannot be maintained because of their methodological limitations, such as age difference between donors and controls, lack of matching of age [36], or selected donor population (donors and recipients with immune-mediated kidney disease are biologically related or African Americans) [13]. Furthermore, a matched cohort study of Reese et al. with a sample of 5152 donors aged > 55 years old showed that there are no differences regarding mortality between older kidney donors and healthy participants [37]. Whether a donor with pre-existing and treated hypertension can be accepted for donation must be considered carefully. One single center study with a low number of older and obese donors demonstrated worse renal function compared to non-obese donors [38]. Especially older donors seem to have an increased obesity-associated risk for hypertension, which cannot be attributed to nephrectomy alone [39, 40]. There are also no data regarding gender-specific consequences of donation. In the US, a trend already indicates that donors are increasingly older, medically complex ill, overweight, and often hypertensive or even have pre-diabetes [39, 41]. Thus, more donors with so-called “isolated medical abnormalities” are accepted.

Studies on the QoL of donors after LKD found similar or slightly higher scores compared to the general population [18, 19, 42]. High QoL is associated with a positive attitude towards the provided donation [9, 29, 32, 42–44], but little is known about risk factors for adverse psychosocial outcomes. Because previous studies were mostly conducted retrospectively, a large variation in the follow-up time after donation (up to 48 years) exists, introducing a potential for recall bias. Many studies were cross-sectional in design allowing no conclusion on the change in QoL after donation. Thus, because of methodological limitations in several prior studies, their results cannot be generalized. The SoLKiD study with its prospective, multicenter design will add to the outcome data of living kidney donors. In view of an ongoing controversial debate about transplantation medicine in Germany, which is likely to further reduce the post mortem organ availability, and also because of the debate on the allowable scope of living donation, the SoLKiD results can provide a valuable database for transplant professionals to facilitate the discussion about potential risks, as well as for patients and their families to help them reach an informed decision about the donation.
